# New parasitoid (Hymenoptera, Chalcidoidea) records of bark beetles (Coleoptera, Curculionidae, Scolytinae) in pine plantations in Bulgaria

**DOI:** 10.3897/BDJ.11.e109325

**Published:** 2023-09-29

**Authors:** Sevdalin Belilov, Ivaylo Todorov, Margarita Georgieva, Georgi Georgiev

**Affiliations:** 1 Forest Research Institute, Bulgarian Academy of Sciences, Sofia, Bulgaria Forest Research Institute, Bulgarian Academy of Sciences Sofia Bulgaria; 2 Institute of Biodiversity and Ecosystem Research, Bulgarian Academy of Sciences, Sofia, Bulgaria Institute of Biodiversity and Ecosystem Research, Bulgarian Academy of Sciences Sofia Bulgaria

**Keywords:** bark beetles, parasitoids, Pteromalidae, Heydeniidae, new records, Bulgaria

## Abstract

**Background:**

In 2020 and 2021, chalcidoid parasitoids (Hymenoptera, Chalcidoidea) of bark beetles in pine (*Pinus* spp.) plantations were studied in Bulgaria. Samples (cuttings of stems and branches) of pine trees infested by bark beetles were collected from seven plantations of *Pinussylvestris* and *P.nigra* in Bulgaria. From each sampling plot, five cuttings were collected and placed in photoeclectors in laboratory conditions (18-22ºC). Emerged bark beetles and parasitoids were regularly gathered and fixed in ethanol.

**New information:**

Six parasitoid species - *Dinotiscuscolon*, *Metacolusazureus*, *M.unifasciatus*, *Rhopalicusquadratus*, *R.tutela* (Chalcidoidea: Pteromalidae) and *Heydeniapretiosa* (Chalcidoidea, Heydeniidae) were reared from five bark beetle hosts (*Ipsacuminatus*, *Pityogenesbistridentatus*, *Pityophthoruspityographus*, *Tomicuspiniperda* and *T.minor*). Amongst them, three species (*H.pretiosa*, *M.azureus* and *R.quadratus*) are recorded as new for Bulgarian fauna.

## Introduction

In the 1950s and 1960s, intensive forest planting has been developed under the process of afforestation on degraded and deforested lands in the lower forest belt in Bulgaria. *Pinusnigra* Arn. and *Pinussylvestris* L. have been the main species used in forest plantations in this region. Increasing the age of plantations in the habitats has led to an increase in moisture deficit, deterioration of the physiological condition of plants and a reduction of their resistance to abiotic and biotic effects (Mirchev et al. 2016). These factors had an adverse effect on forests, especially on the pine monocultures in the lower forest belt (Yangyozov 2010).

In recent decades, forest mortality has increased for many tree species in the countries in the Mediterranean Region due to dry and warm conditions (Allen et al. 2010). In Bulgaria, the tendency for decreasing precipitation during the vegetation period had an adverse effect on forests reducing their resistance to biotic and abiotic factors. This creates a prerequisite for increased development of population density of biotic agents, such as bark beetles or fungal pathogens, especially in pine plantations created outside of their natural range (Yangyozov 2011).

A total of 121 species of bark beetles have been established in Bulgaria and about half of them are associated with pines (*Pinus* spp.) (Doychev 2014). Some of them, mainly representatives of the genera *Ips* and *Tomicus*, appear as destructive pests in pine plantations weakened by various abiotic factors (Mirchev et al. 2016). Their ecology is not well studied, especially with regard to trophic associations with chalcidoid parasitoids (Chalcidoidea, Hymenoptera).

The present study reports new chalcidoid parasitoids of bark beetles for the Bulgarian fauna and information about their impact on host abundance.

## Materials and methods

Our studies of parasitoids (Hymenoptera, Chalcidoidea) of bark beetles were conducted during the years 2020-2022 in seven plantations of *Pinussylvestris* L. and *Pinusnigra* Arn. growing in five localities in Bulgaria. The main characteristics of the studied areas are presented in Table 1.

Samples (cuttings of stems and branches of approximate length 30-35 cm and diameter 12-30 cm for stems and 5-10 cm for branches) of trees attacked by bark beetles were collected in May-August. In each plantation, five trees were examined and sampled.

The material was transported to the Forest Research Institute in Sofia where each cutting was kept in a separate photoeclector at room temperature (18-22ºC). The samples were observed weekly for the emergence of adult hosts or parasitoids.

The emerged bark beetles were identified using the keys of Karaman (1971) and Grüne (1979). Parasitoids were identified following Graham (1969), Gibson (2003) and Tselikh (2010).

The biological material was deposited in the entomological collections of the Forest Research Institute and Institute of Biodiversity and Ecosystem Research in Sofia.

## Checklists

### Checklist

#### Dinotiscus colon

(Linnaeus, 1758)

3434D274-12EF-5A06-BBD6-B695B98A37D1

##### Materials

- **Type status:** Other material. **Occurrence:** recordedBy: S. Belilov leg. [SB]; sex: 1 male; establishmentMeans: Rearing from *Ipsacuminatus*; occurrenceID: C7ED67B3-F891-54F3-9BB7-B797E14D58CA; **Location:** country: Bulgaria; municipality: Elin Pelin; locality: Krushovitsa; verbatimElevation: 697 m; verbatimLatitude: 42.590466; verbatimLongitude: 23.65752; **Event:** startDayOfYear: 06/06/2020; endDayOfYear: 23/07/2020

##### Native status

native

#### Metacolus azureus

(Ratzeburg, 1844)

ED549267-EBE2-5DC2-9E6D-1BFA9227ADA9

##### Materials

- **Type status:** Other material. **Occurrence:** recordedBy: S. Belilov leg. [SB]; sex: 4 males, 6 females; establishmentMeans: Rearing from *Ipsacuminatus*, *Pityogenesbistridentatus*, *Pityophthoruspityographus*, *Tomicuspiniperda*, *Tomicusminor*; occurrenceID: 54F1A671-E15F-525B-9F25-4CB56D1C60C1; **Location:** country: Bulgaria; municipality: Elin Pelin; locality: Krushovitsa; verbatimElevation: 697 m; verbatimLatitude: 42.590466; verbatimLongitude: 23.65752; **Event:** startDayOfYear: 06/06/2020; endDayOfYear: 05/08/2020

##### Native status

native

#### Metacolus unifasciatus

(Foerster, 1856)

038E54BF-4FBF-535C-8C12-F3BAB0E9C983

##### Materials

- **Type status:** Other material. **Occurrence:** recordedBy: S. Belilov leg. [SB]; sex: 11 males, 15 females; establishmentMeans: Rearing from *Ipsacuminatus*, *Pityogenesbistridentatus*, *Pityophthoruspityographus*; occurrenceID: 10BEBAA0-9021-59A5-98EB-45A2B0DCEB98; **Location:** country: Bulgaria; municipality: Elin Pelin; locality: Krushovitsa; verbatimElevation: 697 m; verbatimLatitude: 42.590466; verbatimLongitude: 23.65752; **Event:** startDayOfYear: 06/06/2020; endDayOfYear: 05/08/2020
- **Type status:** Other material. **Occurrence:** recordedBy: S. Belilov leg. [SB]; sex: 2 males, 3 females; establishmentMeans: Rearing from *Ipsacuminatus*; occurrenceID: 899EC99C-99D5-50B7-8790-25FC081F2981; **Location:** country: Bulgaria; municipality: Ihtiman; locality: Mechkovtsi; verbatimElevation: 939 m; verbatimLatitude: 42.55608; verbatimLongitude: 23.747801; **Event:** startDayOfYear: 29/06/2022; endDayOfYear: 05/07/2022

##### Native status

native

#### Rhopalicus quadratus

(Ratzeburg, 1844)

AFB2B233-AF5E-53E1-8666-C5DBA0EDC86F

##### Materials

- **Type status:** Other material. **Occurrence:** recordedBy: S. Belilov leg. [SB]; sex: 13 males, 11 females; establishmentMeans: Rearing from *Ipsacuminatus*, *Tomicuspiniperda*, *Tomicusminor*; occurrenceID: CCF09913-6DDD-57E8-9035-635C44EDAE2C; **Location:** country: Bulgaria; municipality: Elin Pelin; locality: Golema Rakovitsa; verbatimElevation: 651 m; verbatimLatitude: 42.615944; verbatimLongitude: 23.784333; **Event:** startDayOfYear: 06/06/2020; endDayOfYear: 02/09/2020
- **Type status:** Other material. **Occurrence:** recordedBy: S. Belilov leg. [SB]; sex: 46 males, 27 females; establishmentMeans: Rearing from *Ipsacuminatus*, *Pityogenesbistridentatus*, *Pityophthoruspityographus*; occurrenceID: 33D856DE-18EA-50C7-847D-344904C26DD3; **Location:** country: Bulgaria; municipality: Elin Pelin; locality: Krushovitsa; verbatimElevation: 697 m; verbatimLatitude: 42.590466; verbatimLongitude: 23.65752; **Event:** startDayOfYear: 06/06/2020; endDayOfYear: 02/08/2020
- **Type status:** Other material. **Occurrence:** recordedBy: S. Belilov leg. [SB]; sex: 5 males; establishmentMeans: Rearing from *Tomicuspiniperda*, *Tomicusminor*; occurrenceID: 8A07ACFC-6FF9-57FA-982D-FA938C3F726F; **Location:** country: Bulgaria; municipality: Ihtiman; locality: Mechkovtsi; verbatimElevation: 939 m; verbatimLatitude: 42.55608; verbatimLongitude: 23.747801; **Event:** startDayOfYear: 18/06/2021; endDayOfYear: 01/07/2021
- **Type status:** Other material. **Occurrence:** recordedBy: S. Belilov leg. [SB]; sex: 10 males, 18 females; establishmentMeans: Rearing from *Ipsacuminatus*, *Tomicusminor*; occurrenceID: DBF82BC2-E5AF-5816-9C8B-74B09D14EB97; **Location:** country: Bulgaria; municipality: Sofia, Pancharevo region; locality: Dolni Pasarel; verbatimElevation: 889 m; verbatimLatitude: 42.437758; verbatimLongitude: 23.649787; **Event:** startDayOfYear: 11/07/2022; endDayOfYear: 21/07/2022
- **Type status:** Other material. **Occurrence:** recordedBy: S. Belilov leg. [SB]; sex: 5 males, 7 females; establishmentMeans: Rearing from *Ipsacuminatus*; occurrenceID: 2F261A1F-BFE9-5010-84EA-71D08C3C11A4; **Location:** country: Bulgaria; municipality: Ihtiman; locality: Venkovets; verbatimElevation: 828 m; verbatimLatitude: 42.436282; verbatimLongitude: 23.668128; **Event:** startDayOfYear: 06/06/2020; endDayOfYear: 01/07/2020

##### Native status

native

#### Rhopalicus tutela

(Walker, 1836)

E79CC2B2-77A4-5777-8D48-092D4D3CECFF

##### Materials

- **Type status:** Other material. **Occurrence:** recordedBy: S. Belilov leg. [SB]; sex: 3 males, 1 female; establishmentMeans: Rearing from *Tomicuspiniperda*, *Tomicusminor*; occurrenceID: DA9884E5-C355-527C-B4F8-B23A190DC204; **Location:** country: Bulgaria; municipality: Elin Pelin; locality: Golema Rakovitsa; verbatimElevation: 651 m; verbatimLatitude: 42.615944; verbatimLongitude: 23.784333; **Event:** startDayOfYear: 11/07/2022; endDayOfYear: 01/08/2022
- **Type status:** Other material. **Occurrence:** recordedBy: S. Belilov leg. [SB]; sex: 3 males, 3 females; establishmentMeans: Rearing from *Ipsacuminatus*; occurrenceID: 6F8E5C68-164A-563E-8A71-EF2AFD81ECCB; **Location:** country: Bulgaria; municipality: Sofia, Pancharevo region; locality: Dolni Pasarel; verbatimElevation: 833 m; verbatimLatitude: 42.418542; verbatimLongitude: 23.617609

##### Native status

native

#### Heydenia pretiosa

(Förster, 1856)

402C4B0F-6F22-5BB8-9622-933AC163DC2E

##### Materials

- **Type status:** Other material. **Occurrence:** recordedBy: S. Belilov leg. [SB]; sex: 2 males, 18 females; establishmentMeans: Rearing from *Ipsacuminatus*, *Pityogenesbistridentatus*, *Pityophthoruspityographus*; occurrenceID: 92C41B55-3328-50C3-997D-14A77D564FDF; **Location:** country: Bulgaria; municipality: Elin Pelin; locality: Krushovitsa; verbatimElevation: 697 m; verbatimLatitude: 42.590466; verbatimLongitude: 23.65752; **Event:** startDayOfYear: 06/06/2020; endDayOfYear: 31/07/2020
- **Type status:** Other material. **Occurrence:** recordedBy: S. Belilov leg. [SB]; sex: 1 female; establishmentMeans: Rearing from *Tomicuspiniperda*, *Tomicusminor*; occurrenceID: 32F813A2-A298-563E-B2F5-25FCA7EF9A43; **Location:** country: Bulgaria; municipality: Elin Pelin; locality: Golema Rakovitsa; verbatimElevation: 651 m; verbatimLatitude: 42.615944; verbatimLongitude: 23.784333; **Event:** startDayOfYear: 06/06/2020; endDayOfYear: 31/07/2020
- **Type status:** Other material. **Occurrence:** recordedBy: S. Belilov leg. [SB]; sex: 1 male, 1 female; establishmentMeans: Rearing from *Ipsacuminatus*; occurrenceID: 9A29ABAA-EE82-59E5-B617-7465E323CA9D; **Location:** country: Bulgaria; municipality: Ihtiman; locality: Venkovets; verbatimElevation: 828 m; verbatimLatitude: 42.436282; verbatimLongitude: 23.668128; **Event:** startDayOfYear: 11/07/2022; endDayOfYear: 21/07/2022
- **Type status:** Other material. **Occurrence:** recordedBy: S. Belilov leg. [SB]; sex: 2 males, 2 females; establishmentMeans: Rearing from *Ipsacuminatus*, *Tomicusminor*; occurrenceID: 35FB0428-21CA-5C9A-B4E5-EFF0E84F07E2; **Location:** country: Bulgaria; municipality: Sofia, Pancharevo region; locality: Dolni Pasarel; verbatimElevation: 831 m; verbatimLatitude: 42.424683; verbatimLongitude: 23.616527; **Event:** startDayOfYear: 11/07/2022; endDayOfYear: 01/08/2022

##### Native status

native

## Analysis

Six parasitoid species from two chalcidoid families were found to emerge from the tree samples: *Dinotiscuscolon* Linnaeus, 1758, *Metacolusazureus* Ratzeburg, 1844, *Metacolusunifasciatus* Foerster, 1856, *Rhopalicusquadratus* (Ratzeburg, 1844), *Rhopalicustutela* (Walker, 1836) (Chalcidoidea, Pteromalidae) and *Heydeniapretiosa* Förster, 1856 (Chalcidoidea, Heydeniidae) (Table 2).

The parasitoids emerged from five species of bark beetles (Coleoptera, Curculionidae, Scolytinae): *Ipsacuminatus* (Gyllenhal, 1827), *Pityogenesbistridentatus* (Eichhoff, 1878), *Pityophthoruspityographus* (Ratzeburg, 1837), *Tomicuspiniperda* (Linnaeus, 1758) and *Tomicusminor* (Hartig, 1834).

In this study, three species (*H.pretiosa*, *M.azureus* and *R.quadratus*) were newly recorded for the Bulgarian fauna.

During the study period, *R.quadratus* (64.0%) was the most abundant species in the total number of the emerged parasitoids, followed by *M.unifasciatus* (14.0%), *H.pretiosa* (12.6%), *Metacolusazureus* (4.5%) and *Rhopalicustutela* (4.5%), whereas *Dinotiscuscolon* was represented by 0.4%.

The parasitism varied widely in individual species and groups of bark beetles and in different localities: in *I.acuminatus* from 47.8% (Krushovitsa loc.) to 90.5% (Dolni Pasarel loc.); in *T.piniperda* and *T.minor* from 2.1-2.8% (Mechkovtsi loc.) to 91.7% (Rakovitsa loc.); in *P.bistridentatus* and *P.pityographus* from 7.7 to 89.6% (Krushovitsa) (Table 3). It should be emphasised that, in individual cases, both more than one host and more than one parasitoid were isolated from the photoeclector samples.

## Discussion

The chalcidoids found in this study are well known as parasitoids of bark beetles: *Dinotiscuscolon* was previously reared from *Ipsacuminatus*, *Tomicusminor* and *T.piniperda*; *Heydeniapretiosa* – from *I.acuminatus*, *I.typographus*, *T.minor*, *Orthotomicuserosus* (Wollaston, 1857) and *Pityokteinesvorontzowi* (Jakobson, 1895); *Metacolusazureus* – from *I.acuminatus*, *O.erosus*, *Pityogenesconjunctus* (Reitter, 1887), *Pityogeneschalcographus* (Linnaeus, 1761), *T.minor* and *T.piniperda* (Kenis et al. 2004, Wegensteiner et al. 2015); *Metacolusunifasciatus* – from *I.acuminatus*, *I.typographus*, *O.erosus*, *T.minor* and *T.piniperda* (Kenis et al. 2004, Wegensteiner et al. 2015); *Rhopalicusquadratus* – from *I.acuminatus*, *Ipsamitinus* (Eichhoff, 1871), *P.chalcographus*, *Pityokteinescurvidens* (Germar, 1824), *Polygraphuspoligraphus* (Linnaeus, 1758), *T.minor* and *T.piniperda* (Kenis et al. 2004, Wegensteiner et al. 2015); *Rhopalicustutela* – from *Dendroctonusmicans* (Kugelann, 1794), *I.acuminatus*, *I.amitinus*, *Ipsduplicatus* (Sahlberg, 1836), *Ipssexdentatus* (Börner, 1776), *I.typographus*, *P.chalcographus*, *P.curvidens*, *P.poligraphus*, *T.minor* and *T.piniperda* (Kenis et al. 2004, Wegensteiner et al. 2015).

Three chalcidoid species (*Heydeniapretiosa*, *Metacolusazureus* and *Rhopalicusquadratus*) were reported as new for the Bulgarian fauna. One of them, *H.pretiosa*, was recently elevated from Pteromalidae to family rank (Heydeniidae) by Burks et al. (2022).

As concerns the remaining parasitoid species, *Dinotiscuscolon* has been previously recorded from Bulgaria as a parasitoid of *Scolytusamygdali* Guerin, 1847, *Scolytusmali* (Bechstein, 1805) and *Scolytusrugulosus* (Muller, 1818) (Thompson 1954). In addition, another species from the genus, *Dinotiscuseupterus* Walker, 1836, was established as a parasitoid of *Ipstypographus* (Linnaeus, 1758) in Bulgaria (Georgiev and Stojanova 2006).

*Metacolusunifasciatus* has been previously recorded from Bulgaria as a parasitoid of Ovalisia (Palmar) festiva (Linnaeus, 1767) (Coleoptera, Buprestidae) (Ruseva et al. 2020).

*Rhopalicustutela* was first recorded for the Bulgarian fauna by Vidal (1993) and was reported again later as a parasitoid of *I.typographus* by Georgiev and Stojanova (2006).

It can be concluded that the species composition and the population dynamics of chalcidoid parasitoids of bark beetles in pine stands in Bulgaria have not been studied sufficiently in the past. In this study, high levels of host mortalities were registered, but more field observations are necessary to evaluate the actual impact of these natural enemies on the pest populations.

## Supplementary Material

XML Treatment for Dinotiscus colon

XML Treatment for Metacolus azureus

XML Treatment for Metacolus unifasciatus

XML Treatment for Rhopalicus quadratus

XML Treatment for Rhopalicus tutela

XML Treatment for Heydenia pretiosa

## Figures and Tables

**Table 1.** Main characteristics of the studied areas.

№	Locality	Geographical coordinates	Altitude, m a.s.l.	Tree species	Origin	Age, years	Year of study
1	Golema Rakovitsa	N 42.615944, E 23.784333	651	*Pinussylvestris*	Plantation	40	2020
2	Krushovitsa	N 42.590466, E 23.657520	697	*Pinussylvestris*	Plantation	55	2020
3	Mechkovtsi	N 42.556080, E 23.747801	939	*Pinussylvestris*	Plantation	45	2021-2022
4	Venkovets	N 42.436282, E 23.668128	828	*Pinussylvestris*	Plantation	50	2022
5	Dolni Pasarel 1	N 42.424683, E 23.616527	831	*Pinussylvestris*	Plantation	55	2022
6	Dolni Pasarel 2	N 42.437758, E 23.649787	889	*Pinussylvestris*	Plantation	55	2022
7	Dolni Pasarel 3	N 42.418542, E 23.617609	833	*Pinusnigra*	Plantation	55	2022

**Table 2.** Number and relative abundance of different species in the parasitoid complex.

**Species**	**Host**	**Locality**	**Date of collection**	**E mergence** **period**	**Parasitoid number**			**Relative share**, %
					♀♀	♂♂	**Ʃ**	
*Dinotiscuscolon*	*I.acuminatus*	Krushovitsa	05.06.2020	06.06.-23.07.2020	1	0	1	0.4
*Heydeniapretiosa	*I.acuminatus*	Krushovitsa	05.06.2020	06.06.-31.07.2020	11	1	12	12.6
		D. Pasarel 1	11.07.2022	11.07.-01.08.2022	1	0	1	
		Venkovets	11.07.2022	11-21.07.2022	1	1	2	
	*P.bistridentatus*	Krushovitsa	05.06.2020	06.06.-31.07.2020	5	1	6	
	*T.piniperda*, *T.minor*	G. Rakovitsa	05.06.2020	06.06.-31.07.2020	1	0	1	
	*I.acuminatus*, *P.bistridentatus*, *P.pityographus*	Krushovitsa	05.06.2020	06.06.-02.08.2020	2	0	2	
	*T.minor*	D. Pasarel 1	11.07.2022	11-21.07.2022	1	0	1	
		D. Pasarel 3	11.07.2022	11.07.-01.08.2022	1	2	3	
*Metacolusazureus	*I.acuminatus*	Krushovitsa	05.06.2020	06.06.-05.08.2020	1	0	1	4.5
	*P.bistridentatus*	Krushovitsa	05.06.2020	06.06.-23.07.2020	3	0	3	
	*I.acuminatus*, *P.bistridentatus*, *P.pityographus*	Krushovitsa	05.06.2020	06.06.-05.08.2020	2	3	5	
	*T.piniperda*, *T.minor*	Krushovitsa	05.06.2020	06.06.-05.08.2020	0	1	1	
*Metacolusunifasciatus*	*I.acuminatus*	Krushovitsa	05.06.2020	06.06.-05.08.2020	6	4	10	14.0
		Mechkovtsi		29.06.-05.07.2022	3	2	5	
	*P.bistridentatus*	Krushovitsa	05.06.2020	06.06.-23.07.2020	7	5	12	
	*P.bistridentatus*, *P.pityographus*	Krushovitsa	05.06.2020	06.06.-02.08.2020	0	1	1	
	*I.acuminatus*, *P.bistridentatus*, *P.pityographus*	Krushovitsa	05.06.2020	06.06.-02.08.2020	2	1	3	
*Rhopalicusquadratus	*Ipsacuminatus*	G. Rakovitsa	05.06.2020	06.06.-02.09.2020	5	2	7	64.0
		Krushovitsa	05.06.2020	06.06.-05.08.2020	14	15	29	
		D. Pasarel 1	11.07.2022	11.07.-01.08.2022	2	0	2	
		D. Pasarel 2	11.07.2022	11-21.07.2022	9	5	14	
		Venkovets	11.07.2022	11-21.07.2022	7	5	12	
	*P.bistridentatus*	Krushovitsa	05.06.2020	06.06.-23.07.2020	10	29	39	
	*I.acuminatus*, *P.bistridentatus*, *P.pityographus*	Krushovitsa	05.06.2020	06.06.-02.08.2020	3	1	4	
	*T.piniperda*, *T.minor*	G. Rakovitsa	05.06.2020	06.06.-01.07.2020	6	11	17	
	*T.piniperda*, *T.minor*	Krushovitsa	05.06.2020	06.06.-05.08.2020	0	1	1	
	*T.minor*	Mechkovtsi	16.06.2021	18.06.-01.07.2021	2	0	2	
		D. Pasarel 1	11.07.2022	11-21.07.2022	7	5	12	
	*T.minor*, *T.piniperda*	Mechkovtsi	16.06.2021	18.06.-01.07.2021	3	0	3	
*Rhopalicustutela*	*T.piniperda*, *T.minor*	G. Rakovitsa	05.06.2020	06.06.-01.07.2020	1	3	4	4.5
	*I.acuminatus*	D. Pasarel 1	11.07.2022	11.07.-01.08.2022	0	1	1	
	*I.acuminatus*	D. Pasarel 2	11.07.2022	11-21.07.2022	3	2	5	
**Total**					**120**	**102**	**222**	**100.0**

**Table 3.** Parasitism of bark beetles in studied localities.

**Locality**	**Year**	**Parasitoid species**	**Bark beetle host**	**Parasitism**, %
Golema Rakovitsa	2020	*Rhopalicusquadratus*	*Ipsacuminatus*	53.8
		*Heydeniapretiosa*, *Rhopalicusquadratus*, *Rhopalicustutela*	*Tomicuspiniperda*, *Tomicusminor*	91.7
Krushovitsa	2020	*Dinotiscuscolon*, *Heydeniapretiosa*, *Metacolusazureus*, *Metacolusunifasciatus*, *Rhopalicusquadratus*	*Ipsacuminatus*	47.8
		*Heydeniapretiosa*, *Metacolusazureus*, *Metacolusunifasciatus*, *Rhopalicusquadratus*	*Ipsacuminatus*, *Pityogenesbistridentatus*, *Pityophthoruspityographus*	58.3
		*Heydeniapretiosa*, *Metacolusazureus*, *Metacolusunifasciatus*, *Rhopalicusquadratus*	*Pityogenesbistridentatus*	89.6
		*Metacolusunifasciatus*	*Pityogenesbistridentatus*, *Pityophthoruspityographus*	7.7
		*Metacolusazureus*, *Rhopalicusquadratus*	*Tomicuspiniperda*, *Tomicusminor*	14.3
Mechkovtsi	2020	*Rhopalicusquadratus*	*Tomicuspiniperda*	2.8
		*Rhopalicusquadratus*	*Tomicuspiniperda*, *Tomicusminor*	2.1
	2021	*Metacolusunifasciatus*	*Ipsacuminatus*	71.4
Venkovets	2021	*Heydeniapretiosa*, *Rhopalicusquadratus*	*Ipsacuminatus*	66.7
Dolni Pasarel 1	2021	*Heydeniapretiosa*, *Rhopalicusquadratus*, *Rhopalicustutela*	*Ipsacuminatus*	66.7
		*Heydeniapretiosa*, *Rhopalicusquadratus*	*Tomicusminor*	37.1
Dolni Pasarel 2	2021	*Rhopalicusquadratus*, *Rhopalicustutela*	*Ipsacuminatus*	90.5
Dolni Pasarel 3	2021	*Heydeniapretiosa*	*Tomicusminor*	18.8
